# Supplementary material for: Repeatability of automated body composition measurement on low dose chest CT in male subjects
Source: PLoS One. 2026 Apr 17;21(4):e0332004. doi: 10.1371/journal.pone.0332004 (PMC13089885; doi:10.1371/journal.pone.0332004)
Supplement: S1 Appendix — (DOCX) [file pone.0332004.s001.docx]

# S1 Appendix: Training of Truncated FOV method’s models

The models used in the *truncated FOV* method were provided without weights, and thus required training using images and segmentations before they could be used in our study. To train the models, we used a subset of 1031 NELSON participants from UMC Utrecht (University Medical Center Utrecht) whose scans were previously manually segmented [21]. This dataset has no overlap with the dataset used to evaluate repeatability.

## Cross-sectional subset with ground truth segmentations

This subset of 1031 subjects contains 1031 images that have previously been manually segmented into fat and muscle. It was used to train the slice selection model and segmentation model of the *truncated FOV* method. The fat and muscle segmentations were made using in house developed software. The scans were reconstructed to have a slice thickness of 3.1 mm at 1.4 mm increment to reduce noise. During the segmentation process, the observer selected the first CT slice above the aortic arch, after which voxels in that slice containing fat and skeletal muscles were visually identified and manually segmented using brushing and region growing. Predefined attenuation ranges based on the different Hounsfield Units (HU) of these tissues were used. For visceral fat this was -150 to -50 HU, for SAT -190 to -30 HU and for muscles -29 to 150 HU [22]. Inaccuracies were manually corrected.

In this cross-sectional data (N=1031), the median age was 61.5 (25%-75%, 58.6 – 65.6) years, all were male, 53% were current smokers. For 849 subjects BMI was available (mean±SD, 26.3±3.4).

## Modification of the training loss

The original research used a slightly modified form of the Dice loss for training the segmentation network (Equation (1)).

$-\sum_{i} \frac{2*{Area of Overlap}_{i}}{{{Predicted Area}_{i} + True Area}_{i}}$ **(1)**

For gradient descent optimization, this formula is equivalent to Equation 2.

$\sum_{i} 1-\frac{2*{Area of Overlap}_{i}}{{{Predicted Area}_{i} + True Area}_{i}}$ **(2)**

However, we found that it had a tendency to prioritize one tissue type over all others during training of the model. This lead to the output segmentations only containing a single tissue type. For that reason the loss was modified to put more weight on difficult classes (Equation (3)).

$\sum_{i} {(1-\frac{2*{Area of Overlap}_{i}}{{Predicted Area}_{i}+ {True Area}_{i}})}^{2}$ **(3)**

## Training of the models

The models were trained using the previously mentioned cross-sectional subset. The 1031 subjects from this dataset were randomly split into a 727 (70%) subject training set, 208 (20%) subject validation set, and a 104 (10%) subject test set. Training parameters such as learning rate and optimizer were the same as in the original work. The Dice coefficients of the test set were calculated to evaluate model performance.


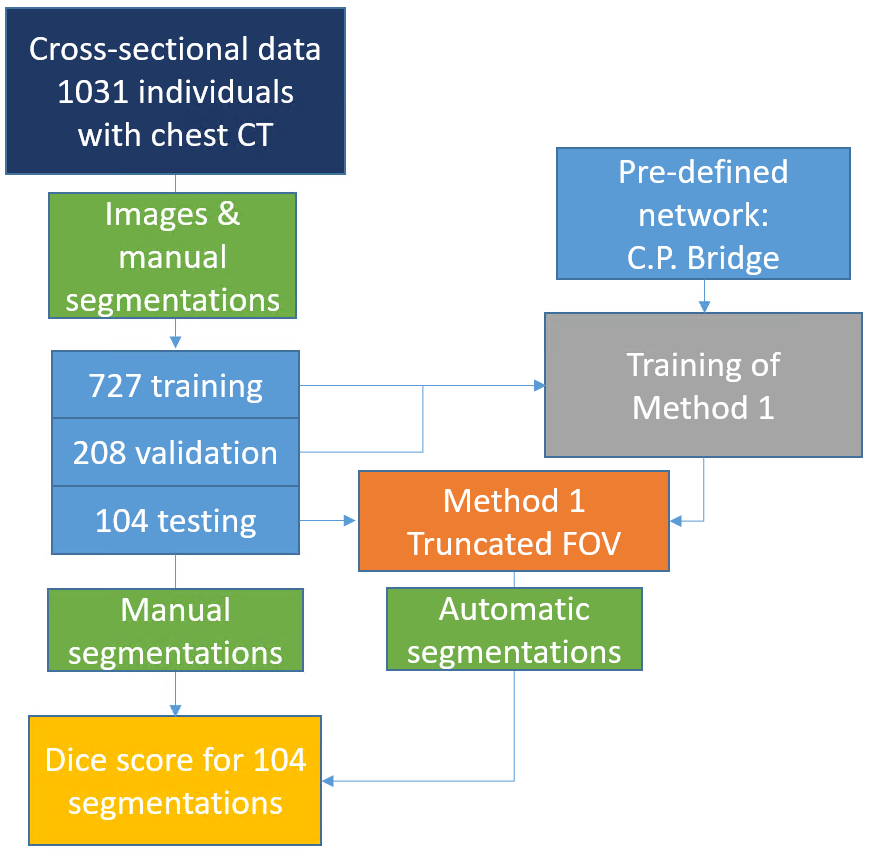


Figure S1: Flowchart of datasets and methods used in training. Details of datasets and methods are explained elsewhere. Pre-defined network means that a pre-existing network shape is used, but without weights.

#### Calculating tissue area

To help enhance the consistency of the pipeline when determining tissue area, segmentations are created not just for the selected slice, but also for two slices on both sides of it. This results in five slices segmented per subject. For each of the tissue types, the area (cm²) and the average radiodensity (HU) for a single slice were calculated by taking the mean across the five segmented slices. This is different from the *extended FOV* method, which segments and measures three single slices at T5, T8, and T10.

## Performance of the re-trained models

The *truncated FOV* model, when re-trained with our data, achieved a slice selection RMSE of 3.1 mm. Evaluating the segmentation performance yielded a mean Dice coefficient of 0.95 over the test set of 104 subjects and 104 images. The mean Dice of skeletal muscle and SAT were 0.95 and 0.96 respectively.
