# Supplementary figures and images for: Repeatability of automated body composition measurement on low dose chest CT in male subjects
Source: PLoS One. 2026 Apr 17;21(4):e0332004. doi: 10.1371/journal.pone.0332004 (PMC13089885; doi:10.1371/journal.pone.0332004)

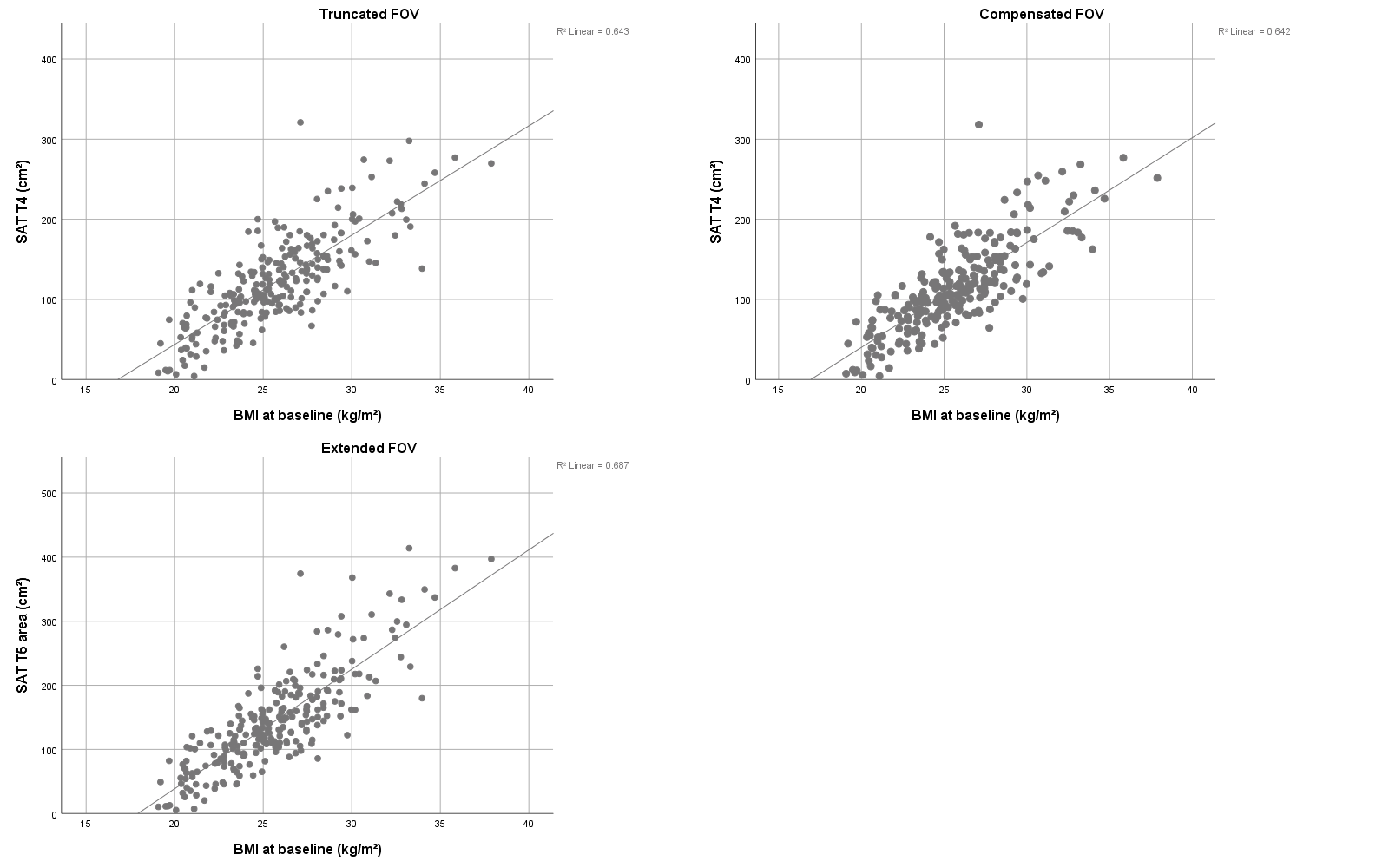

Supplement: S2 Fig — Correlation plots between BMI and SAT for the truncated, compensated, and extended FOV methods. (TIF) [file pone.0332004.s002.tif]
