# Supplementary material for: Repeatability of automated body composition measurement on low dose chest CT in male subjects
Source: PLoS One. 2026 Apr 17;21(4):e0332004. doi: 10.1371/journal.pone.0332004 (PMC13089885; doi:10.1371/journal.pone.0332004)
Supplement: S3 Appendix — (DOCX) [file pone.0332004.s003.docx]

# S3 Appendix: Details on the registration process for *compensated FOV*

Registration was performed with python using ITK 5.3rc4.post2 and itk-elastix 0.14.2. We used a SimilarityTransform to perform translation, scaling, and rotation. A default pixel value of -1000 was used to reflect the normal HU of empty space in a CT image. After registering the CT images, the same transform that was applied to the moving CT image was applied to the segmentation corresponding to the moving CT image, with two important differences: The default pixel value was set to 0, the background label, and the final B-spline interpolation order was set to 0. This B-spline interpolation order was used to prevent non-integer tissue labels. The full code for the registration and area calculation steps can be found at https://github.com/SAOBunk/Compensated-FOV.


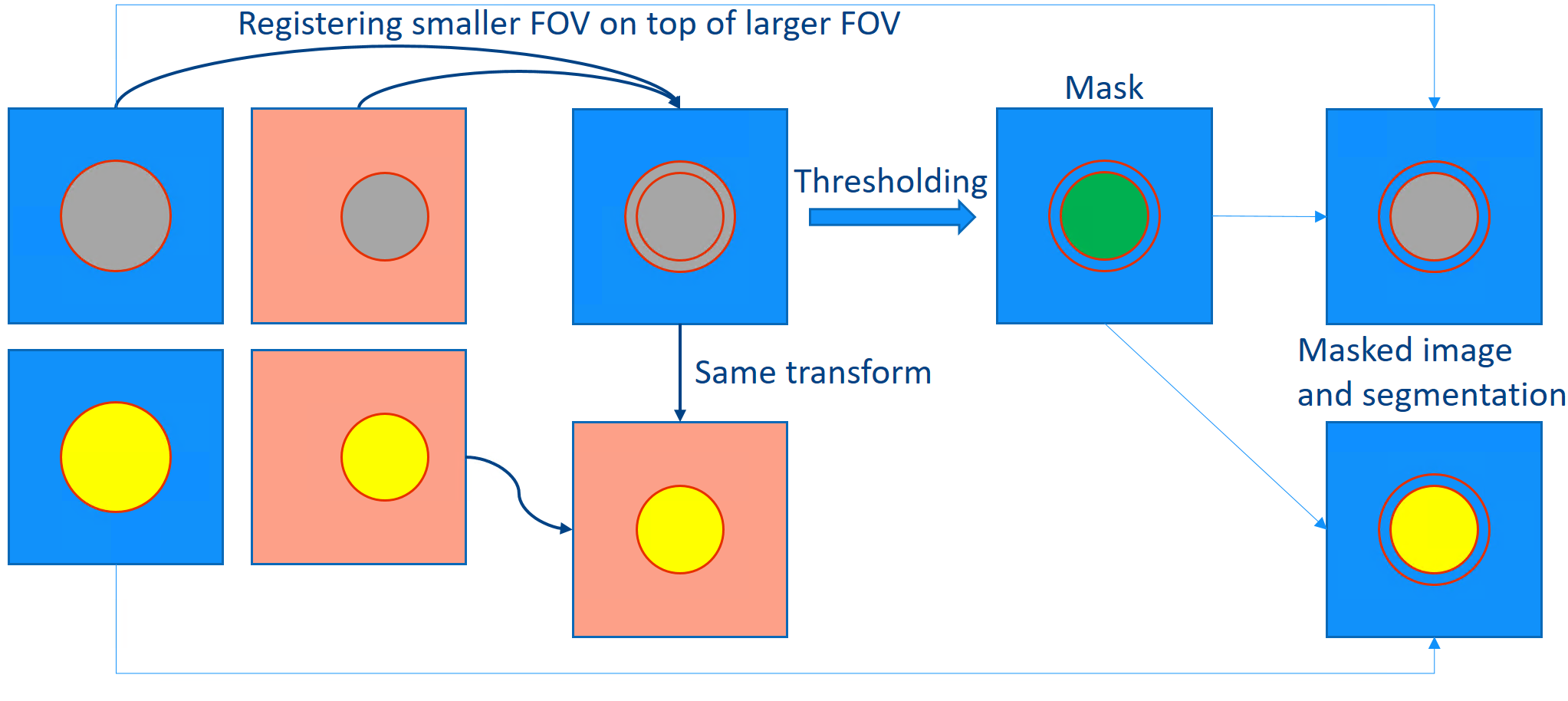


Figure A: Schematic representation of registration process. Gray: CT Image. Yellow: Segmentation. Green: inclusion mask. Orange background indicates the moving image. Size of circles indicates size of image FOV.
