# Supplementary material for: Repeatability of automated body composition measurement on low dose chest CT in male subjects
Source: PLoS One. 2026 Apr 17;21(4):e0332004. doi: 10.1371/journal.pone.0332004 (PMC13089885; doi:10.1371/journal.pone.0332004)
Supplement: S4 Appendix — (DOCX) [file pone.0332004.s004.docx]

# S4 Appendix: Differences in measurements based on scan kVp

| Table A. Comparison of scan areas based on scan kVp. | | | | | | | | | | |
| --- | --- | --- | --- | --- | --- | --- | --- | --- | --- | --- |
|  | Mean Area (cm²) | | | | | |  |  |  |  |
|  | First Scan Moment | | | Second Scan Moment | | | Non-matching kVp (n=103) | | Matching kVp (n=459) | |
|  | 120 kVp (n=273) | 140 kVp (n=289) | *p*-value | 120 kVp (n=266) | 140 kVp (n=296) | *p*-value | Agreement *p*-value | ICC | Agreement *p*-value | ICC |
| Truncated FOV | | | | | | | | | | |
| Skeletal Muscle | 194 ± 35 | 230 ± 31 | <0.001 | 193 ± 36 | 229 ± 31 | <0.001 | 0.048 | 0.849 [0.776, 0.898] | 0.541 | 0.925 [0.910, 0.938] |
| SAT | 111 ± 60 | 152 ± 54 | <0.001 | 109 ± 57 | 153 ± 58 | <0.001 | 0.679 | 0.953 [0.931, 0.968] | 0.949 | 0.970 [0.964, 0.975] |
| Compensated FOV | | | | | | | | | | |
| Skeletal Muscle | 190 ± 35 | 225 ± 31 | <0.001 | 189 ± 35 | 225 ± 31 | <0.001 | 0.188 | 0.959 [0.939, 0.972] | 0.837 | 0.975 [0.970, 0.979] |
| SAT | 103 ± 55 | 143 ± 53 | <0.001 | 103 ± 54 | 145 ± 54 | <0.001 | 0.044 | 0.980 [0.971, 0.987] | 0.477 | 0.988 [0.985, 0.990] |
| Extended FOV | | | | | | | | | | |
| Skeletal Muscle T5 | 195 ± 36 | 228 ± 32 | <0.001 | 194 ± 36 | 225 ± 30 | <0.001 | 0.005 | 0.924 [0.883, 0.950] | 0.198 | 0.946 [0.935, 0.955] |
| SAT T5 | 128 ± 74 | 181 ± 73 | <0.001 | 127 ± 72 | 183 ± 75 | <0.001 | 0.539 | 0.972 [0.959, 0.981] | 0.741 | 0.986 [0.983, 0.988] |
| SAT=Subcutaneous Fat. Mean Area reported ± SD. SD = Standard Deviation. *p*-value=independent samples T-test *p*-value comparing the area values for 120 kVp to those for 140 kVp. Non-matching kVp=The subjects who were scanned at a different kVp for the first scan than for the second scan. Matching kVp=The subjects who were scanned at the same kVp for both scans. Agreement *p*-value=Paired samples T-test *p*-value of area measured by method in first scan compared to second scan. ICC = intraclass correlation coefficient of mean area for the first and second scan moments, values in square brackets are the 95% confidence interval. | | | | | | | | | | |

| Table B. Comparison of scan radiodensities based on scan kVp. | | | | | | | | |
| --- | --- | --- | --- | --- | --- | --- | --- | --- |
|  | First Scan Moment | | | Second Scan Moment | | |  |  |
|  | 120 kVp (n=273) | 140 kVp (n=289) | *p*-value | 120 kVp (n=266) | 140 kVp (n=296) | *p*-value | *p*-value non-matching kVp (n=103) | *p*-value matching kVp (N=459) |
| Truncated FOV | | | | | | | | |
| Muscle Mean (HU) | 40.1 ± 4.2 | 36.0 ± 3.8 | <0.001 | 40.1 ± 4.1 | 35.3 ± 3.4 | <0.001 | 0.121 | 0.114 |
| Muscle SD Mean (HU) | 33.1 | 33.4 | 0.314 | 33.0 | 33.0 | 0.972 | 0.311 | 0.224 |
| Compensated FOV | | | | | | | | |
| Muscle Mean (HU) | 40.0 ± 4.2 | 36.0 ± 3.7 | <0.001 | 40.0 ± 4.1 | 35.2 ± 3.4 | <0.001 | 0.107 | 0.118 |
| Muscle SD Mean (HU) | 33.9 | 34.3 | 0.179 | 34.0 | 34.0 | 0.881 | 0.517 | 0.892 |
| Extended FOV | | | | | | | | |
| Muscle Mean (HU) | 27.7 ± 5.2 | 23.1 ± 4.1 | <0.001 | 27.8 ± 5.1 | 22.5 ± 4.1 | <0.001 | 0.321 | 0.023 |
| Muscle SD Mean (HU) | 39.5 | 39.4 | 0.64 | 39.5 | 39.2 | 0.053 | 0.935 | 0.418 |
| HU = Hounsfield Units; SD = Standard Deviation. Muscle Mean reported ± SD. Muscle SD Mean refers to the mean of all scans' standard deviation. The '*p*-value' columns compare the HU values for 120 kVp to those for 140 kVp using a independent samples T-test. The '*p*-value non-matching/matching kVp’ columns compare the radiodensity during the first scan moment to that of the second scan moment using a paired samples T-test. | | | | | | | | |





Figure A: Bland-Altman plots for scan pairs with different kVp values (n=103) of difference in area between the two scans as a percentage of the mean area of those two scans. The vertical mean line is mean area in cm2 across all scans. The horizontal mean line is the mean difference between two scans.





Figure B: Bland-Altman plots for scan pairs with the same kVp values (n=459) of difference in area between the two scans values as a percentage of the mean area of those two scans. The vertical mean line is mean area in cm2 across all scans. The horizontal mean line is the mean difference between two scans.
