# Supplementary material for: Repeatability of automated body composition measurement on low dose chest CT in male subjects
Source: PLoS One. 2026 Apr 17;21(4):e0332004. doi: 10.1371/journal.pone.0332004 (PMC13089885; doi:10.1371/journal.pone.0332004)
Supplement: Attachment — Submitted filename: Response to Reviewers.docx [file pone.0332004.s005.docx]

# Response to Reviewers

We would like to thank the Editor and Reviewer for their careful examination of our manuscript and their valuable feedback. We note the editor and reviewer have several concerns. The following are comment-by-comment replies and excerpts of the changes made. The changes in the excerpts are highlighted in red. We hope the changes to our manuscript satisfy the Editor’s and Reviewer’s concerns.

## **Editor’s comments:**

*1. When submitting your revision, we need you to address these additional requirements.*

*Please ensure that your manuscript meets PLOS ONE's style requirements, including those for file naming. The PLOS ONE style templates can be found at*

*https://journals.plos.org/plosone/s/file?id=wjVg/PLOSOne_formatting_sample_main_body.pdf and*

*https://journals.plos.org/plosone/s/file?id=ba62/PLOSOne_formatting_sample_title_authors_affiliations.pdf*

**Author’s response:** We have studied the style templates and file naming requirements and updated the manuscript style, references, and file names to match.

*2. We note that the grant information you provided in the ‘Funding Information’ and ‘Financial Disclosure’ sections do not match.*

*When you resubmit, please ensure that you provide the correct grant numbers for the awards you received for your study in the ‘Funding Information’ section.*

**Author’s response:** Our apologies for incorrectly reporting the grant number. The correct grant number is 9037. This grant number has also been provided in the updated Funding Statement. It may also be important to note that the Dutch Cancer Society is the English name for KWF Kankerbestrijding.

*3. Thank you for stating in your Funding Statement:*

*This work is supported by funding from the Dutch Cancer Society, Siemens Healthineers, and by the Ministry of Economic Affairs and Climate Policy by means of the Public–Private Partnerships Allowance made available by the Top Sector Life Sciences & Health to stimulate public–private partnerships. Funding information for this article has been deposited with the Crossref Funder Registry*

*RV recieved the grant under project number 9037 from the Dutch Cancer Society.*

*https://www.kwf.nl/onderzoek/onderzoeksdatabase/longkankerscreening-op-maat-met-multidata-voorspelmodellen*

*The funders had no role in study design, data collection and analysis, decision to publish, or preparation of the manuscript.*

*Please provide an amended statement that declares *all* the funding or sources of support (whether external or internal to your organization) received during this study, as detailed online in our guide for authors at http://journals.plos.org/plosone/s/submit-now.  Please also include the statement “There was no additional external funding received for this study.” in your updated Funding Statement.*

*Please include your amended Funding Statement within your cover letter. We will change the online submission form on your behalf.*

**Author’s response:** Our funding statement declares all funding and sources of support. We have updated our funding statement to indicate the first author’s PhD project was supported by the Dutch Cancer Society grant. We have updated our Funding Statement to include “There was no additional external funding received for this study.”.

This work is supported by funding from the Dutch Cancer Society, Siemens Healthineers, and by the Ministry of Economic Affairs and Climate Policy by means of the Public–Private Partnerships Allowance made available by the Top Sector Life Sciences & Health to stimulate public–private partnerships. Funding information for this article has been deposited with the Crossref Funder Registry.

RV received the indicated grant under project number 9037 from the Dutch Cancer Society. <https://www.kwf.nl/onderzoek/onderzoeksdatabase/longkankerscreening-op-maat-met-multidata-voorspelmodellen>. The PhD project of the first author was supported by this grant.

The funders had no role in study design, data collection and analysis, decision to publish, or preparation of the manuscript.

There was no additional external funding received for this study.

*4. Thank you for stating the following in the Competing Interests section:*

*I have read the journal's policy and the authors of this manuscript have the following competing interests:*

*RV declares a relationship with the following company: Siemens Healthineers*

*The other authors of this manuscript declare no relationships with any companies, whose products or services may be related to the subject matter of the article.*

*We note that one or more of the authors are employed by a commercial company: Siemens Healthineers.*

*a. Please provide an amended Funding Statement declaring this commercial affiliation, as well as a statement regarding the Role of Funders in your study. If the funding organization did not play a role in the study design, data collection and analysis, decision to publish, or preparation of the manuscript and only provided financial support in the form of authors' salaries and/or research materials, please review your statements relating to the author contributions, and ensure you have specifically and accurately indicated the role(s) that these authors had in your study. You can update author roles in the Author Contributions section of the online submission form.*

*Please also include the following statement within your amended Funding Statement.*

*“The funder provided support in the form of salaries for authors, but did not have any additional role in the study design, data collection and analysis, decision to publish, or preparation of the manuscript. The specific roles of these authors are articulated in the ‘author contributions’ section.”*

*If your commercial affiliation did play a role in your study, please state and explain this role within your updated Funding Statement.*

*b. Please also provide an updated Competing Interests Statement declaring this commercial affiliation along with any other relevant declarations relating to employment, consultancy, patents, products in development, or marketed products, etc.*

*Within your Competing Interests Statement, please confirm that this commercial affiliation does not alter your adherence to all PLOS ONE policies on sharing data and materials by including the following statement: "This does not alter our adherence to  PLOS ONE policies on sharing data and materials.” (as detailed online in our guide for authors http://journals.plos.org/plosone/s/competing-interests) . If this adherence statement is not accurate and  there are restrictions on sharing of data and/or materials, please state these. Please note that we cannot proceed with consideration of your article until this information has been declared.*

*Please include both an updated Funding Statement and Competing Interests Statement in your cover letter. We will change the online submission form on your behalf.*

**Author’s response:** There is some misunderstanding. None of the authors are employed by Siemens Healthineers. RV works at a university medical center. The relationship with Siemens Healthineers is limited to institutional research grants and speaker fees, and not employment. We have updated the Competing Interests section to make this distinction clear. We have included this amended Competing Interests section in our cover letter.

I have read the journal's policy and the authors of this manuscript have the following competing interests:

RV declares receiving institutional grants and speaker fees from the following company: Siemens Healthineers.

This does not alter our adherence to PLOS ONE policies on sharing data and materials.

The other authors of this manuscript declare no relationships with any companies, whose products or services may be related to the subject matter of the article.

*5. We note that you have indicated that there are restrictions to data sharing for this study. For studies involving human research participant data or other sensitive data, we encourage authors to share de-identified or anonymized data. However, when data cannot be publicly shared for ethical reasons, we allow authors to make their data sets available upon request. For information on unacceptable data access restrictions, please see http://journals.plos.org/plosone/s/data-availability#loc-unacceptable-data-access-restrictions.*

*Before we proceed with your manuscript, please address the following prompts:*

*a) If there are ethical or legal restrictions on sharing a de-identified data set, please explain them in detail (e.g., data contain potentially identifying or sensitive patient information, data are owned by a third-party organization, etc.) and who has imposed them (e.g., a Research Ethics Committee or Institutional Review Board, etc.). Please also provide contact information for a data access committee, ethics committee, or other institutional body to which data requests may be sent.*

*b) If there are no restrictions, please upload the minimal anonymized data set necessary to replicate your study findings to a stable, public repository and provide us with the relevant URLs, DOIs, or accession numbers. Please see http://www.bmj.com/content/340/bmj.c181.long for guidelines on how to de-identify and prepare clinical data for publication. For a list of recommended repositories, please see https://journals.plos.org/plosone/s/recommended-repositories. You also have the option of uploading the data as Supporting Information files, but we would recommend depositing data directly to a data repository if possible.*

*Please update your Data Availability statement in the submission form accordingly*.

**Author’s response:** In response to point a), we regret we cannot share the data publicly due to privacy concerns and restrictions imposed by the NELSON Data Access Board. We have updated our Data Availability statement to align fully with PLOS One requirements and now also include contact information for the NELSON Data Access Board. In response to point b), due to the restrictions imposed by the NELSON Data Access Board we cannot upload an anonymized data set.

Data cannot be shared publicly because of privacy concerns. Data are available from the NELSON Data Access Board (contact via https://umcgresearchdatacatalogue.nl/all/resources/NELSON) for researchers who meet the criteria for access to confidential data.

*6. One of the noted authors is a group or consortium “NELSON-POP study group”. In addition to naming the author group, please list the individual authors and affiliations within this group in the acknowledgments section of your manuscript. Please also indicate clearly a lead author for this group along with a contact email address.*

**Author’s response:** Our apologies for misreporting the name of the consortium in the author list. We have updated the author list to correctly refer to the NELSON-POP consortium. We have also indicated the lead author and added contact information for the lead author.

Changes made in line 11:

NELSON-POP consortium^^^

Changes made in line 19:

^^^Membership of the NELSON-POP consortium is provided in the Acknowledgements.

Changes made in line 342-344:

*All members of the NELSON-POP consortium:*

**Lead author:** Rozemarijn Vliegenthart, MD, PhD, University Medical Center Groningen, University of Groningen, Groningen, The Netherlands**. Email:** r.vliegenthart@umcg.nl

*7. Please amend either the abstract on the online submission form (via Edit Submission) or the abstract in the manuscript so that they are identical.*

**Author’s response:** We have amended the abstract on the online submission form to align with the abstract in the manuscript.

*8. Please upload a new copy of Figures 5, 6, S2, S4 and S5, as the detail is not clear. Please follow the link for more information:  https://journals.plos.org/plosone/s/figures*

**Author’s response:** Thank you for bringing these deficiencies to our attention. We have used PLOS’s free figure tool NAAS to re-process the images. We have uploaded these new versions.

*9. Please include captions for your Supporting Information files at the end of your manuscript, and update any in-text citations to match accordingly. Please see our Supporting Information guidelines for more information: http://journals.plos.org/plosone/s/supporting-information.*

**Author’s response:** We have updated our supporting information files, updated in-text citations, and included a Supporting Information files section at the end of the manuscript. In the Supporting Information files section we have

added titles and captions.

Changes made in line 450-455:

# Supporting Information

**S1 Appendix. Training of Truncated FOV method’s models.**

**S2 Fig. Correlation of BMI with SAT*.*** Correlation plots between BMI and SAT for the truncated, compensated, and extended FOV methods.

**S3 Appendix. Details on the registration process for *compensated FOV*.**

**S4 Appendix. Differences in measurements based on scan kVp.**

*10. If the reviewer comments include a recommendation to cite specific previously published works, please review and evaluate these publications to determine whether they are relevant and should be cited. There is no requirement to cite these works unless the editor has indicated otherwise.*

**Author’s response:** No reviewer has requested to cite specific previously published works.

**Additional Editor Comments:**

*This study evaluates and compares the repeatability of three automated methods for measuring body composition—specifically skeletal muscle area and subcutaneous adipose tissue area—from low-dose chest CT scans in a longitudinal setting. The manuscript is concise, well-organized, and clearly presented. Although it does not introduce significant technical novelty, it represents a valid investigation that offers meaningful insights for the medical field, supported by a large dataset. That said, I have several technical questions that require clarification:*

*First, I have concerns regarding the radiodensity discrepancies observed among the three methods. The extended FOV method underwent notably different preprocessing compared to the other two methods. It remains unclear how the authors could confidently claim—and rule out—that these discrepancies were not attributable to preprocessing. In particular, the fact that discrepancies persisted when the extended FOV method was applied to datasets from the other two methods suggests to me that its processing pipeline may introduce systematic errors. As the authors noted, downsampling could indeed lead to segmentation issues.*

**Author’s response:** Our apologies for being unclear. We intended to convey that when the *extended FOV* preprocessing steps (downsampling from 512x512 to 256x256 and clipping to -150HU to 150HU) were applied as post-processing steps to the scans and segmentations used in and created by the *truncated/compensated FOV* methods, the HU measurements they provide did not change. That is why we concluded that the preprocessing did not affect the HU measurements. We have clarified this in the discussion.

Changes made in line 276-279:

When the preprocessing steps of the *extended FOV* method named in a) and b) were applied as post-processing steps to the CT scans and segmentations from the *truncated/compensated FOV* method, the radiodensity measurements provided by the *truncated/compensated* FOV method did not change.

*Second, regarding the study population, all CT data are from male participants. The rationale for including only males—or whether only male data were available—is not explained. Further details on the population selection criteria are needed.*

**Author’s response:** Unfortunately so few females with short-term follow-up scans were available that it would have introduced unintended variability in our technical evaluation of the effect of FOV cutoff on measurement repeatability. Therefore we selected only men. We have clarified this in the methods.

Changes made in line 90-95:

This subset contained a group of 562 male subjects with two CT scans taken at an interval of 3-4 months. In the NELSON trial, only 13.9% of the available short-term follow-up CT data from University Medical Center Utrecht was from females, inclusion of which would have introduced unintended variability in the technical evaluation of the effect of FOV cutoff of tissues on measurement repeatability. Therefore, we only report on male subjects.

*Third, some findings appear obvious and contribute little novelty. For example, in line 222, the statement “We also found that scans collected at 120 kVp … compared to those collected at 140 kVp” reflects a well-understood principle that higher kVp reduces attenuation and thus lowers radiodensity measurements. This finding seems unnecessary and not directly relevant to the core aims of the study.*

**Author’s response:** Indeed, this is an expected finding. We have corrected our wording to reflect this.

Changes made in line 236:

As expected, we found that scans collected at 120 kVp had a slightly higher measured radiodensity compared to those collected at 140 kVp.

*Finally, certain statements are overly vague. For instance, line 95 states: “Participants with higher weight were scanned with a higher kVp to ensure similar image quality as the participants with lower weight.” However, it is unclear how many participants were affected and what specific kVp values were used in these cases.*

**Author’s response:** Our apologies, our wording was unclear in this paragraph. We meant to indicate that participants scanned at 140 kVp were scanned at that tube voltage to maintain similar image quality as those scanned at 120 kVp. All participants were scanned at either 120 kVp or 140 kVp. We have rewritten this section to make this information clear to readers. Information on the number of participants scanned at 120/140 kVp is available in S4 Appendix. During the first scan moment 273 subjects were scanned at 120 kVp and 289 subjects at 140 kVp. During the second scan moment 266 subjects were scanned at 120 kVp and 296 subjects at 140 kVp.

Changes made in line 105-109:

The following settings were used: fixed 30 mAs and 120 kVp for participants weighing 80 kg or less and 140 kVp for those weighing more than 80 kg. Participants weighing 80kg or more were scanned with 140 kVp to ensure similar image quality to the participants weighing 80kg or less, who were scanned with 120 kVp.

*Minor formality issues:*

*Line 207, cm2 is not current format of the unit.*

*Footnote of Table 2. SAT=Subcutaneous Fat. Please stay consistent, SAT = subcutaneous adipose tissue.*

**Author’s response:** Thank you for bringing these matters to our attention. They have been corrected.

Changes made in line: 220-222

**Fig 5. Bland-Altman plots of difference in area between two scans.** The difference is displayed as a percentage of the mean area of those two scans. The vertical mean line is mean area in cm^2^ across all scans. The horizontal mean line is the mean difference between two scans.

Changes made in Table 2:

| Table 2. Measurement data of the 562 subjects for the *Truncated*, *Compensated*, and *Extended FOV* methods. | | | | | | | |
| --- | --- | --- | --- | --- | --- | --- | --- |
|  |  | Mean Area (cm²) | Mean Difference (%) | Lower limit (%) | Upper Limit (%) | Agreement *p*-value | ICC |
| Truncated FOV | Skeletal Muscle | 212 | 0.7 | -18.9 | 20.4 | 0.125 | 0.914 [0.898, 0.927] |
|  | SAT | 132 | 0.5 | -37.3 | 38.3 | 0.806 | 0.967 [0.962, 0.972] |
| Compensated FOV | Skeletal Muscle | 208 | 0.3 | -11.1 | 11.6 | 0.421 | 0.973 [0.968, 0.977] |
|  | SAT | 125 | -0.4 | -30.2 | 29.3 | 0.126 | 0.987 [0.984, 0.989] |
| Extended FOV | Skeletal Muscle T5 | 211 | 0.7 | -16.7 | 18.2 | 0.017 | 0.942 [0.932, 0.951] |
|  | SAT T5 | 156 | 0.4 | -29.1 | 29.9 | 0.995 | 0.984 [0.981, 0.986] |
| SAT=Subcutaneous Adipose Tissue. Upper and lower limit indicate the 95% agreement boundaries, with the value being the percentage difference between a subject’s two scans relative to the mean. Agreement *p*-value=Paired samples T-test *p*-value of area measured by method in first scan compared to second scan. ICC = intraclass correlation coefficient, values in square brackets are the 95% confidence interval. | | | | | | | |

**Reviewers' comments:**

Reviewer's Responses to Questions

**Comments to the Author**

1. Is the manuscript technically sound, and do the data support the conclusions?

The manuscript must describe a technically sound piece of scientific research with data that supports the conclusions. Experiments must have been conducted rigorously, with appropriate controls, replication, and sample sizes. The conclusions must be drawn appropriately based on the data presented.

Reviewer #1: Yes

2. Has the statistical analysis been performed appropriately and rigorously?

Reviewer #1: Yes

3. Have the authors made all data underlying the findings in their manuscript fully available?

The [PLOS Data policy](https://eur05.safelinks.protection.outlook.com/?url=http%3A%2F%2Ftrack.editorialmanager.com%2FCL0%2Fhttp%3A%252F%252Fwww.plosone.org%252Fstatic%252Fpolicies.action%2523sharing%2F1%2F010f019be118e177-d9726948-a338-4a38-95dd-8a8e9f149d0a-000000%2FAKLoLWxp5mOSISmAFjowLj2jqGXOBIv9_c0ARL8oxdU%3D247&data=05%7C02%7Cs.a.o.bunk-2%40umcutrecht.nl%7Cfca15b090e4e4be9f0e408de58fed473%7Cdcdf4a3dd0c04a6394cf781981249be5%7C0%7C0%7C639046048719433083%7CUnknown%7CTWFpbGZsb3d8eyJFbXB0eU1hcGkiOnRydWUsIlYiOiIwLjAuMDAwMCIsIlAiOiJXaW4zMiIsIkFOIjoiTWFpbCIsIldUIjoyfQ%3D%3D%7C0%7C%7C%7C&sdata=IHXrufBhHeUbd6b2i0u%2FBtKK9juRSVD%2FYewhW6LOGJY%3D&reserved=0) requires authors to make all data underlying the findings described in their manuscript fully available without restriction, with rare exception (please refer to the Data Availability Statement in the manuscript PDF file). The data should be provided as part of the manuscript or its supporting information, or deposited to a public repository. For example, in addition to summary statistics, the data points behind means, medians and variance measures should be available. If there are restrictions on publicly sharing data—e.g. participant privacy or use of data from a third party—those must be specified.

Reviewer #1: No

4. Is the manuscript presented in an intelligible fashion and written in standard English?

PLOS ONE does not copyedit accepted manuscripts, so the language in submitted articles must be clear, correct, and unambiguous. Any typographical or grammatical errors should be corrected at revision, so please note any specific errors here.

Reviewer #1: Yes

5. Review Comments to the Author

Please use the space provided to explain your answers to the questions above. You may also include additional comments for the author, including concerns about dual publication, research ethics, or publication ethics. (Please upload your review as an attachment if it exceeds 20,000 characters)

**Reviewer #1:**

*The manuscript addresses the repeatability of automated body composition measurements on longitudinal chest CT scans. Specifically, it evaluates three approaches for compensating field-of-view (FOV) changes between scans: truncated FOV, compensated FOV, and extended FOV. Using a substantial cohort of 562 male participants from the NELSON lung cancer screening trial with baseline and short-term follow-up scans, the authors compare these methods for skeletal muscle and subcutaneous adipose tissue (SAT) area measurements. Repeatability was assessed using Bland–Altman plots, paired t-tests, and intraclass correlation coefficients (ICC).*

*Key findings indicate that the compensated FOV method achieved the highest repeatability but underestimated SAT due to exclusion of non-overlapping regions. Extended FOV produced measurements closest to actual anatomy and exhibited the lowest segmentation error rates, though it introduced a small systematic difference in skeletal muscle area between scans. The authors conclude that extended FOV may be preferable for longitudinal analyses where absolute measurement values are critical.*

**Author’s response:** Thank you for your feedback on our manuscript.

*1. Population Limitation: The study includes only male participants, limiting generalizability. Anatomical differences in women may affect segmentation accuracy. Have the authors considered validating their approach on publicly available datasets that include female subjects (e.g.,* [*https://atm22.grand-challenge.org/dataset/*](https://atm22.grand-challenge.org/dataset/)*)?*

**Author’s response:** Thank you for your suggestion. It is correct that anatomical differences in females may affect segmentation accuracy and therefore the repeatability of measurements. However, the limitation we experienced is that only 13.9% of the NELSON trial’s short-term follow-up CT scan data from University Medical Center Utrecht was from females, inclusion of which would have introduced varability that would detract from the technical evaluation of the effect of FOV cutoff on the three different methods evaluated. Even datasets such as the NLST or the dataset suggested by you do not include short-term follow-up low-dose CT data for females. It is therefore required that large enough short-term follow-up low-dose CT datasets are created for females before any repeatability analyses can be done. We have more clearly outlined this limitation in our methods.

Changes made in line 90-95:

This subset contained a group of 562 male subjects with two CT scans taken at an interval of 3-4 months. In the NELSON trial, only 13.9% of the available short-term follow-up CT data from University Medical Center Utrecht was from females, inclusion of which would have introduced unintended variability in the technical evaluation of the effect of FOV cutoff of tissues on measurement repeatability. Therefore, we only report on male subjects.

*2. Assumption of Stability: The assumption of stable body composition over 3–4 months may not universally hold, despite limited supporting evidence cited.*

**Author’s response:** Thank you for your feedback. We have expanded our explanation of this limitation to indicate that body composition may not be stable overall in every population.

Changes made in line 319-323:

Secondly, we assume no systematic changes in body composition over a 3-4 month interval, as lifestyle programs were no part of the screening trial (outside of advice to stop smoking). However, this assumption may not universally hold in other populations, especially those in which interventions are undertaken. Nevertheless, our results may underestimate repeatability, which could be slightly better with shorter scan interval.

*3. Slice Selection Variability: Comparisons between methods are weakened by non-identical slice selection across approaches.*

**Author’s response:** Thank you for your feedback. We have noted this as a limitation and additionally explain that we do not expect this to affect the comparisons within methods.

Changes made in line 323-326:

Thirdly, though care was taken to compare similar levels in the chest, the two methods did not compare the exact same slice, possibly reducing the strength of the comparison between methods. However, it is unlikely this affected the comparisons within methods.

*4. Radiodensity Discrepancy: The observed differences in radiodensity measurements between extended FOV and other methods are noted but not fully explained, leaving uncertainty regarding clinical interpretation.*

**Author’s response:** Thank you for your comment. We have added a sentence to explain that we expect there to not be any clinical implications resulting from this systematic difference in radiodensity measurements. Please see also our response to the editor’s comment on the same topic, where we explain that the discrepancy in radiodensity is not an effect of the pre-processing steps of the *extended FOV* method, as when those same pre-processing steps are applied as post-processing steps to the scans and segmentations from *truncated/compensated FOV* no changes in radiodensity measurements were observed. Our full explanation is in lines 270-287.

Changes made in line 276-279:

When the preprocessing steps of the *extended FOV* method named in a) and b) were applied as post-processing steps to the CT scans and segmentations from the *truncated/compensated FOV* method, the radiodensity measurements provided by the *truncated/compensated* FOV method did not change.

Changes made in line 283-287:

The *truncated FOV* segmentation contains many more holes than the *extended FOV* segmentation. As each method is internally consistent, we do not expect the differences in radiodensity measurements between methods to have any clinical implications, provided any clinical decision thresholds created are calibrated to each method individually.

*5. Computational Efficiency: The manuscript would benefit from a discussion on computational efficiency and integration into clinical workflows. Specifically, what is the trade-off between computational time and accuracy across the best- and worst-performing methods?*

**Author’s response:** Thank you for your suggestion. We have added a discussion on this topic to our manuscript. We did not find that any method had a significant computational efficiency advantage over any other and that all methods performed the analyses rapidly.

Changes made in line 302-310:

For implementation into clinical workflows the time required by each method to perform its measurements is also of interest. Sometimes there is a trade-off between computational time and accuracy, where high performing methods require longer to perform the analysis. However, none of the three methods we evaluated differed significantly in the time required to perform the measurements. The *compensated FOV* method required more computation than the *truncated FOV* method due to the need to also register the images, but this additional time cost was negligible. The *truncated FOV* method and *extended FOV* method required similar amounts of time to process data, despite the *extended FOV* method performing the additional step of FOV extension. For all methods, averaged across all scans processed, processing a single scan took less than a minute.
